# Beyond catheter tip and radiofrequency lesion delivery: the role of robotics in ablation of ventricular tachycardia

**DOI:** 10.1007/s12471-015-0737-y

**Published:** 2015-08-04

**Authors:** L.J. de Vries, F. Zijlstra, T. Szili-Torok

**Affiliations:** Department of Clinical Electrophysiology, Erasmus Medical Center, Rotterdam, The Netherlands

Catheter ablation of ventricular tachycardia (VT) has become an important and increasingly performed treatment [1]. The use of remote magnetic navigation (RMN) for this procedure has been shown to have several advantages compared with manual catheter navigation (MCN) [2]. Since RMN was introduced in 2003 the most obvious advantage of RMN when compared with MCN is safety. Increased safety for both operators and patients is achieved by shortening fluoroscopy time and by reducing complications due to catheter flexibility [3]. So far, there has not been a single report of myocardial perforation. Additionally, after an initial learning curve has been completed, the use of RMN most likely shortens procedure time [4], may prevent operator fatigue and helps to reach the parts of the heart that are less easily accessible during mapping.

In the current issue of the Netherlands Heart Journal, Wu et al. [5] present their meta-analysis on RMN vs. MCN for the ablation of VT. They included four non-randomised studies and conclude that acute and long-term success rates for VT ablation are equal between RMN and MCN. However, complication rates, procedure and fluoroscopy times are favourable for RMN.

Their conclusion adds to the aforementioned findings for improved safety and at least a non-inferior success rate. In fact, the only limitation of RMN that remains in experienced teams is the cost of the equipment which can be up to 2 million € [6]. However, the long-term benefits of this installation should be taken into account when assessing these expenses [6].

Looking at success rate, the authors have been cautious in their interpretation. Several studies have in fact demonstrated a better outcome compared with MCN in VT ablation [4, 7]. What could be a possible explanation for this variable outcome?

As RMN is a relatively new and evolving method, procedural outcomes can be further improved when sufficient experience is obtained. Only a limited group of operators could acquire the appropriate level of skill due to the cost of the system and a consequently limited availability. This could potentially result in an operator-dependent success rate variability and also brings up another point related to the financial aspect. Most of the centres able to afford the installation are academic centres, dealing with a very specific patient population. This means the procedures performed with RMN will be those in patients with a more complex pathology, which in turn may result in an underestimation of treatment success. Finally, because of the limited availability of RMN technology, present studies are mostly non-randomised. Non-significant trends in success rates favourable to RMN, as seen in Dinov et al. [8], may in the future prove significant in randomised trials.

Notably, the papers by Bauernfeind et al. and Szili-Torok et al., both used in the current report, showed superiority to MCN in acute success rates in structurally normal hearts but equal results in structural heart disease [4, 7]. This raises the issue of how RMN can improve success rates. Is it related to the improved manoeuvrability of the catheter tip: tip delivery, or due to the constant type of tissue tip contact which improves radiofrequency lesion formation: lesion delivery? The data from of the above-mentioned manuscripts as well as this meta-analysis indirectly suggest that tip delivery efficiency may be superior using RMN while lesion delivery should be equally good when compared with MCN. RMN is theoretically more suitable for reaching difficult locations because of the flexible nature of the catheter in combination with the possibility of more accurate positioning.

What are the future perspectives? An important step that could definitively make RMN favourable to MCN will be the utilisation of contact force measuring ablation catheters for magnetic navigation and ablation. Optimal electrode—tissue contact has been previously demonstrated to be of great importance for lesion formation [9] and has already been shown to improve clinical outcome in the treatment of atrial fibrillation [10]. When this technique becomes available for RMN and reaches its full potential (new combined parameters, involvement of cardiac imaging for real time visualisation of lesion formation) it might revolutionise treatment of ventricular and supraventricular arrhythmias with RMN and might prove pivotal in achieving long-term success in VT ablation.

## Funding

None.

## Conflict of interests

None declared.
